# Dental Medicine

**Published:** 1884-02

**Authors:** 


### BIBLIOGRAPHICAL.

---

DENTAL MEDICINE, A MANUAL OF DENTAL MATERIA MEDICA AND THERAPEUTICS, for Practitioners and Students. By Ferdinand J. S. Gorgas, A. M., M. D., D. D. S., Editor of "Harris' Principles and Practice of Dentistry," "Harris' Medical and Dental Dictionary," Professor of the Principles of Dental Science, Dental Surgery, etc., in the University of Maryland Dental Department. Publishers: P. Blakiston, Son & Co., Philadelphia, 1884.

This work has been prepared by the authur in deference to many requests from former pupils, and has been compiled from lectures delivered by him in dental institutions during the past twenty-five years, and notes obtained from the standard works on Materia Medica and Therapeutics, and also from personal experience as a dental practitioner and teacher.

While the author claims the credit of the compilation, he does not claim originality in the sources, derivations, medical properties and action of the various articles of dental materia medica which are given in this work.

His intention has been to present not alone his own ideas as to the particular application of remedies, but also those of well known and acknowledged authorities, and in such a manner as may be of service to the dental student in acquiring a knowledge of this important branch of his profession ; hence nothing has been presented in this work that, in the author's opinion, is not applicable to dental practice, and that will not be of benefit to the dental student.

The dental formulary comprises many valuable combinations, and credit has been given, in every case where it was possible, to the authors of the different preparations.

The necessity for an American work of this kind has long been apparent, and after years of delay and promises the author gratefully dedicates this work to his former pupils in the dental institutions with which he has been and is now connected in the capacity of a teacher. The following comprise the table of contents :

Introduction and Definitions and General Remarks : Action of Medicinal Substances: Important Points in Diagnosing Affections of the Mouth : Characteristic Indications of the Tongue ; Abbreviations with Latin and English Terms : Approximate Measurements : Fineness of Powder : Weights and Measures : Metric or French Decimal System of Weights and Measures : Rules for Regulating Doses : Table of Doses of all Official Medicines, Expressed in Terms of Both the Apothecaries, and the Decimal Metric System of Weights and Measures : Poisons—Symptoms and Antidotes : The Pulse : Pulsation per Minute at Various Ages : Respiration at Various Ages : Thermometers : Table of Elementary Substances : Table of the Solubility of Chemicals in Water and Alcohol : Classification of Medicinal Substances : Definitions of the Various Classes of Medicinal Substances : Forms in which Medicinal Substances are Employed : Source, Derivation, Medical Properties and Action, and Therapeutic Uses of Medicinal Substances Employed in Dental Practice ; Together with their Dental Uses and Application : Administration of General Anæsthetic Agents : The Dangers of Anæsthesia : Preventive Measures Against the Dangers of Anæsthesia Treatment of Dangerous Symptoms of Anæsthesia : Methods of Resuscitation—Sylvester's Method—Hall's Ready Method : Local Anæsthesia : Topical Remedies : The Endermic Method.

The Hypodermic Method : Setons and Issues : General Bloodletting : Local Bloodletting : Periods for the Eruption of the Teeth: Electricity as a Therapeutic Means in the Treatment of Disease, etc., etc., price of work $3. For sale by al' Booksellers and Dental Depots.

